# Chitosan nanoparticles synthesis caught in action using microdroplet reactions

**DOI:** 10.1038/srep22260

**Published:** 2016-02-29

**Authors:** Vivek Kamat, Dhananjay Bodas, Kishore Paknikar

**Affiliations:** 1 Nanobioscience, Agharkar Research Institute, GG Agarkar Road, Pune 411 004 India

## Abstract

The ionic gelation process for the synthesis of chitosan nanoparticles was carried out in microdroplet reactions. The synthesis could be stopped instantaneously at different time points by fast dilution of the reaction mixture with DI water. Using this simple technique, the effect of temperature and reactant concentrations on the size and distribution of the nanoparticles formed, as a function of time, could be investigated by DLS and SEM. Results obtained indicated very early (1–5 s) nucleation of the particles followed by growth. The concentration of reactants, reaction temperature as well as time, were found to (severally and collectively) determine the size of nanoparticles and their distribution. Nanoparticles obtained at 4 °C were smaller (60–80 nm) with narrower size distribution. Simulation experiments using Comsol software showed that at 4 °C ‘droplet synthesis’ of nanoparticles gets miniaturised to ‘droplet-core synthesis’, which is being reported for the first time.

Chitosan nanoparticles are biocompatible, relatively non-toxic, biodegradable, and cationic in nature^1^,^2^. Thus, they are well accepted in biomedical applications such as drug delivery^3^,^4^,^5^. Ionic gelation is the most commonly used method for synthesising chitosan nanoparticles^6^. In this method, chitosan precursors are cross-linked using sodium tripolyphosphate (TPP). The method typically yields large sized (100–300 nm) particles with a high degree of polydispersity. Even though ionic gelation is a widely used method and factors governing the size and dispersivity of chitosan nanoparticles (such as the concentration of reactants, temperature, pH, and the level of deacetylation) are well known^7^ our basic understanding of the process at mechanistic level is poor. In the ionic gelation process, TPP crosslinks randomly oriented chitosan molecules, which, in turn, are connected to other similarly cross-linked moieties. Such intra- and inter- molecular cross-linking is rather uncontrolled and leads to polydispersity in the synthesised preparation. Here, we hypothesised that using confined reaction volumes of microdroplets and at preset temperatures; it should be possible to exert good control over the kinetics of nanoparticles synthesis if the reactions could be arrested rapidly at desired time points. Such an approach could enable time-lapsed capture of the synthesis process and provide us an opportunity to understand it at a more fundamental level. Thus, in our microdroplet experiments, synthesis reactions were arrested at varying time points to monitor the nucleation and growth of developing nanoparticles using DLS and SEM. For this purpose a simple yet novel technique, viz. fast dilution of the reaction mixture with DI water was employed. In a separate experiment, we had determined that with a 500-fold dilution, the reaction pH increased sharply from 2.84 ± 0.04 to 5.50 ± 0.05. Such a rise in pH could exert a strong endothermic effect^8^,^9^,^10^, resulting in extensive (90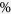) de-protonation of chitosan^11^. Dilution could also result in increased intra-molecular distances, reducing the possibility of molecular interaction. The synthesis of chitosan nanoparticles tends to be arrested by DI water dilution owing to these reasons.

## Results and Discussion

**Figure 1:**
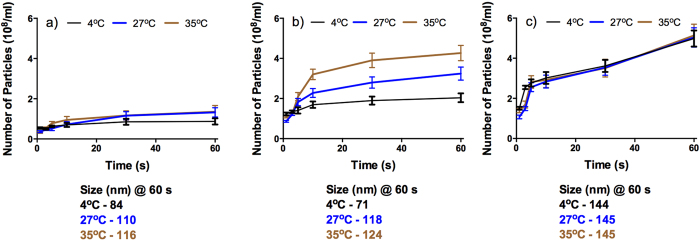
Time- and temperature- dependent change in the number of nanoparticles synthesized using different chitosan concentrations (mg/mL), viz. (a) 0.2; (b) 0.4 and (c) 0.8. Data below the figures detail the sizes of synthesized nanoparticles at 60 s.

It is evident from the data presented in Fig. 1a–c, that with an increase in the concentration of chitosan from 0.2 to 0.8 mg/mL a concomitant increase in the number of nanoparticles formed could be seen. The number of the hyphen should appear after nano (nano-particles) also increased with rising reaction temperature and time. Nanoparticles of smallest size (71 nm) were obtained when the reaction was carried out with 0.4 mg/mL chitosan at 4 °C. The number of nanoparticles was maximum (5.16E8) with 0.8 mg/mL chitosan (at 35 °C). The narrowest size distribution of the particles was seen when the chitosan concentration was 0.4 mg/mL and the reaction was performed at 4 °C (Fig. 3c). It is clear from these data that an interplay of factors like temperature, time and concentration of reactants determines the size, distribution and the number of particles formed.

Interestingly, the number of nanoparticles formed in the initial few seconds (1–5 s) was higher at 4 °C than at 27 °C and 35 °C, irrespective of the chitosan concentration used. It was also seen that the magnitude of difference in the number of nanoparticles formed was highest when the chitosan concentration used was 0.4 mg/mL. Therefore, in further studies chitosan concentration was maintained at 0.4 mg/mL while the reaction temperatures were varied. The time-lapsed capture of the nanoparticles synthesis process was achieved by arresting the reactions at different time points and imaging the preparation by scanning electron microscopy (SEM).

**Figure 2:**
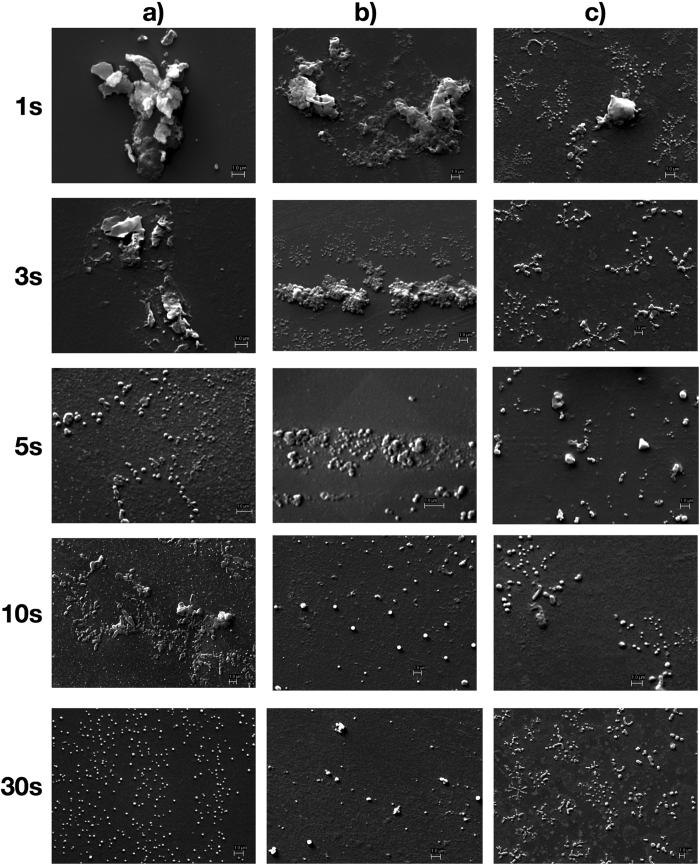
SEM of chitosan nanoparticles captured at varying time points and temperatures. (**a**) 4 °C (**b**) 27 °C and (**c**) 35 °C. The images were captured at 10 kV with a working distance of 9.5 mm.

SEM images presented in Fig. 2a show that at 4 °C, after 1 s, the chitosan polymer was seen in the crystalline state, which rapidly formed a large number of small and monodispersed particles at 3 s. This observation indicated that the nucleation phase was initiated between 1–3 s, followed by growth phase. The image after 10 s (Fig. 2a) shows fully-grown chitosan nanoparticles. At 27 °C (Fig. 2b), unreacted chitosan was seen in 1 s, along with very few nanoparticles. At 3 s, nucleation of the nanoparticles was initiated on the surface of chitosan polymer (seen as bright spots decorating the polymer). At 5 s, the conversion of the polymer to well defined nascent nanoparticles was visible which subsequently underwent growth. In the reactions carried out at 35 °C (Fig. 2c), conversion of chitosan polymer to nanoparticles was instantaneous. Simultaneous and seamless nucleation and growth followed thereafter. Such apparently uncontrolled growth of nanoparticles formed agglomerates at 35 °C resulting in a larger size (130–190 nm) and broader size distribution, as seen after 10 s. On the other hand, nanoparticles obtained at 4 °C were smaller (60–80 nm) and with narrower size distribution.

To discern the mechanism of chitosan nanoparticles synthesis at 4 °C, simulation studies were carried out using Comsol Multiphysics software. The studies focused on understanding the heat and solute transport across the droplet.

**Figure 3:**
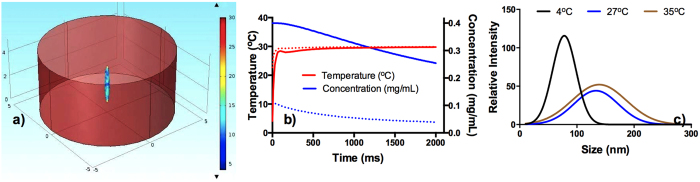
(**a**) Simulation model of the droplet and the surrounding medium. (**b**) Simulation profile showing the variation of temperature and reactant concentration as a function of time. Dotted and continuous line represent temperature and concentration at the droplet periphery and the core respectively; (**c**) Effect of temperature on the size distribution of chitosan nanoparticles (chitosan concentration used 0.4 mg/mL).

Figure 3a shows a simulation model of a (chitosan + TPP) droplet diluted with DI water. The properties of chitosan + TPP were allocated to the droplet whereas DI water properties were ascribed to the surrounding medium. To study heat transfer, the temperature values assigned to the droplet, and the surrounding medium were 4 °C and 30 °C, respectively. Assigned reactant concentrations of the droplet and the medium were 0.4 mg/mL and 0 mg/mL, respectively. Heat and solute transfers were simulated over 0 to 5 s (the experimentally observed period crucial in the formation of chitosan nanoparticles) and were monitored at the core and periphery of the droplet.

The results of simulation studies are shown in Fig. 3b. Chitosan droplet (at 4 °C) upon dilution with DI water (at 30 °C), experiences a sharp rise in the temperature of the droplet core as well as the periphery in 100 ms. At this stage, the chitosan concentration at the droplet core was 0.4 mg/mL, which gradually decreased to 0.25 mg/mL in 2000 ms. In contradistinction, the chitosan concentration in the droplet periphery dropped instantaneously to 0.1 mg/mL, and lowered further to 0.05 mg/mL in 2000 ms.

The ultrafast dilution of the reactants (owing to diffusion) in the droplet periphery as observed here precludes any possibility of fresh nucleation and/or growth. On the other hand, steep rise the in the droplet core temperature along with low molecular mobility favour faster reaction kinetics. A gradual decrease in chitosan concentration in the core over a period of 2000 ms suggests well-controlled nucleation and growth, leading to the smaller size of nanoparticles formed with a narrower distribution. In short, at 4 °C the droplet synthesis translates into further miniaturised and refined droplet-core synthesis. The experimental validation of these simulation results showed that smaller size and narrower size distribution is indeed obtained at 4 °C as compared to 27 °C and 35 °C (Fig. 3c).

This study provides a critical insight into ionic gelation process of chitosan nanoparticles formation, caught in action, for the first time. It is evident from the data presented that the chitosan nanoparticles nucleate very early (1–5 s) and quickly enter the growth phase. The study shows that the concentration of reactants, temperature, and time, severally and collectively determine the fate of the synthesis process in terms of the size and distribution of particles formed. For example, obtaining smaller sized nanoparticles could be at the expense of distribution and/or number of particles. Our simulation experiments point to a new concept, i.e. the quality of nanoparticles relies on the temperature dependent mechanistic pathway rather than the temporal endpoint. Finally, these studies indicate that when it comes to obtaining superior quality polymeric nanoparticles, the secret lies at the core of the droplet!

## Methods

Chitosan (CS) from crab shell (molecular weight 150000 kD, degree of deacetylation 75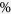) was procured from Sigma-Aldrich, USA. TPP and glacial acetic acid were purchased from Loba Chemie, India and Qualigens, India, respectively. Freshly prepared, deionized (DI) water (pH 6.12 ± 0.03) was used as a diluent. Different sets of reactions were performed where chitosan and TPP concentrations were altered in the range of 0.2–0.8 mg/mL and 0.08–0.32 mg/mL, respectively keeping chitosan:TPP ratio constant (2.5:1). The reaction temperatures used were 4 °C, 27 °C and 35 °C.

Briefly, two (1 *μ*L) droplets of chitosan and TPP were sequentially aspirated using an electronic pipette (Thermo Scientific Novus Finnpipette), allowed to react in the pipette tip and the reactions stopped at varying times (1, 3, 5, 10, 30 and 60 s) by 500-fold dilution with DI water. The number of nanoparticles formed and their sizes were determined by dynamic light scattering (DLS) method using Nanosight LM10 system (Malvern, UK). The nanoparticles formed at different times were also visualized by scanning electron microscopy (Zeiss EVO MA15, UK).

## Additional Information

**How to cite this article**: Kamat, V. *et al.* Chitosan nanoparticles synthesis caught in action using microdroplet reactions. *Sci. Rep.* **6**, 22260; doi: 10.1038/srep22260 (2016).
